# Informative Power Evaluation of Clinical Parameters to Predict Initial Therapeutic Response in Patients with Advanced Pleural Mesothelioma: A Machine Learning Approach

**DOI:** 10.3390/jcm11061659

**Published:** 2022-03-16

**Authors:** Raffaella Massafra, Annamaria Catino, Pia Maria Soccorsa Perrotti, Pamela Pizzutilo, Annarita Fanizzi, Michele Montrone, Domenico Galetta

**Affiliations:** 1 Struttura Semplice Dipartimentale di Fisica Sanitaria, I.R.C.C.S. Istituto Tumori “Giovanni Paolo II”, Viale Orazio Flacco 65, 70124 Bari, Italy; r.massafra@oncologico.bari.it; 2 Struttura Semplice Dipartimentale di Oncologia Medica per la Patologia Toracica, I.R.C.C.S. Istituto Tumori “Giovanni Paolo II”, Viale Orazio Flacco 65, 70124 Bari, Italy; a.catino@oncologico.bari.it (A.C.); p.pizzutilo@oncologico.bari.it (P.P.); m.montrone@oncologico.bari.it (M.M.); galetta@oncologico.bari.it (D.G.); 3 Struttura Semplice Dipartimentale di Radiologia, I.R.C.C.S. Istituto Tumori “Giovanni Paolo II”, Viale Orazio Flacco 65, 70124 Bari, Italy; pia.perrotti@libero.it

**Keywords:** pleural mesothelioma, initial therapeutic response, early prediction, machine learning, clinical parameters

## Abstract

Malignant pleural mesothelioma (MPM) is a rare neoplasm whose early diagnosis is challenging and systemic treatments are generally administered as first line in the advanced disease stage. The initial clinical response may represent a useful parameter in terms of identifying patients with a better long-term outcome. In this report, the initial therapeutical response in 46 patients affected with advanced/unresectable pleural mesothelioma was investigated. The initial therapeutic response was assessed by CT scan and clinical examination after 2–3 treatment cycles. Our preliminary evaluation shows that the group of patients treated with regimens including antiangiogenetics and/or immunotherapy had a significantly better initial response as compared to patients only treated with standard chemotherapy, exhibiting a disease control rate (DCR) of 100% (95% IC, 79.40–100%) and 80.0% (95% IC, 61.40–92.30%), respectively. Furthermore, the therapeutic response was correlated with the disease stage, blood leukocytes and neutrophils, high albumin serum levels, and basal body mass index (BMI). Specifically, the patients with disease stage III showed a DCR of 95.7% (95% IC, 78.1–99.9%), whereas for disease stage IV the DCR decreased to 66.7% (95% IC, 34.9–9.1%). Moreover, a better initial response was observed in patients with a higher BMI, who reached a DCR of 96.10% (95% IC, 80.36–99.90%). Furthermore, in order to evaluate in the predictive power of the collected features a multivariate way, we report the preliminary results of a machine learning model for predicting the initial therapeutic response. We trained a state-of-the-art algorithm combined to a sequential forward feature selection procedure. The model reached a median AUC value, accuracy, sensitivity, and specificity of 77.0%, 75%, 74.8%, and 83.3%, respectively. The features with greater informational power were gender, histotype, BMI, smoking habits, packs/year, and disease stage. Our preliminary data support the possible favorable correlation between innovative treatments and therapeutic response in patients with unresectable/advanced pleural mesothelioma. The small sample size does not allow concrete conclusions to be drawn; nevertheless, this work is the basis of an ongoing study that will also involve radiomics in a larger dataset.

## 1. Introduction

Malignant pleural mesothelioma (MPM) is a rare neoplasm associated with asbestos exposure. Its incidence reached a peak in the early 1990s, although the mortality rate continued to rise [1,2].

In most patients, the disease is diagnosed at an advanced stage, so systemic chemotherapy represents the frontline standard of care although only with a palliative intent. This is generally able to achieve a partial regression or stable disease lasting 6 months on average, although in 15–20% of cases, this can exceed 1 year [2,3].

Recently, immune checkpoint inhibitors (ICIs), combined with antiangiogenetic agents, have emerged as promising treatments [4,5,6,7] and a number of clinical trials have explored their efficacy in these patients [8,9,10,11].

Achieving an early response to chemotherapy represents a major challenge for the efficacy of the treatment in the medium and long term. However, the therapeutical response of mesothelioma depends on multiple factors, such as the histological type (there are three variants: epithelioid, biphasic, and sarcomatoid, with different prognoses and responses to treatments), the extent of the disease, the age and general condition of the patient, and the presence of comorbidities.

With regard to prognostic factors, a number of clinical parameters have been investigated in order to better identify subgroups with different outcomes and to select patient candidates for different therapeutic approaches [12,13].

In fact, the risk assessment has so far been mainly based on the histology and disease stage, limiting the prognostic evaluation, which could be improved by the integration of biological parameters. Recently, the role of the tumor immune microenvironment in pleural mesothelioma development and progression has been highlighted [14].

Hence, the clinicopathological and molecular features could be useful to better define the prognosis of these patients and improve the treatment choice.

Recent studies focused on predicting disease survival of patients with malignant mesothelioma and identifying the prognostic risk and risk factors. Research regarding a reliable prediction model to better stratify subgroups at different risks has led to numerous studies evaluating clinical-biological risk scores and machine learning approaches [15,16,17].

Automated support systems could be a valuable tool to support clinicians in planning and monitoring treatment. Nevertheless, the literature regarding automated models for the early prediction of response to therapy is scarce. The aim of this study was to define the clinical features of these patients and identify a possible correlation between the prognosis and the therapeutic outcome. A statistical correlation between some clinical characteristics and the initial therapeutic response in patients undergoing first-line treatment for advanced/unresectable pleural mesothelioma was investigated. Furthermore, in this report, we describe the preliminary results of a machine learning approach for predicting the initial response to chemotherapy, in order to quantify the informative power of the collected characteristics with respect to the proposed objective. Specifically, the model is used to evaluate how well the baseline clinical features are able to predict the response to therapy measured by radiological criteria. A well-known machine learning algorithm was trained on retrospectively collected data in combination with a sequential forward procedure to select an optimal subset of features. This tool could allow for the early identification of patients that will not demonstrate a favorable initial response to therapy, allowing clinicians to appropriately guide the planning of therapeutic treatment.

## 2. Bibliographical Background

Malignant mesothelioma has raised great interest in the last few years and the main fields of application include etiology, risk factors, epidemiology, and prognosis [18,19]. Regarding this topic, the aid of artificial intelligence techniques is increasingly widespread, allowing for more efficient and functional data analysis to define personalized medicine models [20,21]. Indeed, Alam et al. published a framework proposal for the extraction of knowledge concerning clinical, radiological, and histopathological prognostic factors, on the basis of machine learning for the early diagnosis of malignant mesothelioma [21]; furthermore, the same authors used machine learning and data mining techniques to identify risk factors [22,23].

In addition, recent studies developed automated models for the volumetric assessment of pleural mesothelioma, with the aim of reducing operator-dependent measurement [24].

## 3. Materials and Methods

### 3.1. Materials

From July 2017 to October 2021, we collected clinical data and CT scan images of 46 consecutive patients affected by unresectable pleural mesothelioma who had undergone first-line systemic treatment. A total of 40 patients demonstrated an initial partial response or stable disease upon reassessment, whereas 6 patients exhibited progressive disease.

The study was approved by the Institutional Review Board of Istituto Tumori ‘Giovanni Paolo II’ Bari, Italy (Approval Code: 24269/21).

Patients’ characteristics are shown in Table 1. The initial therapeutic response was evaluated by both clinical examination and by CT scan with mRECIST/RECIST 1.1 criteria [25] at baseline and after 2–3 treatment cycles.

The following clinical features were collected: age at diagnosis, gender, performance status ECOG, body mass index (BMI), comorbidities, asbestos exposure/occupational risk, smoking habits, packs/year, histotype, disease stage, presence of pleural effusion, type of 1st line treatment, leukocytes (Leuk), neutrophils (Ne), lymphocytes (Ly), neutrophil- lymphocyte ratio (NLR), hemoglobin (Hb), platelets (PLT), LDH, and albumin serum levels.

### 3.2. Statistical Analysis and Classification Models

For each characteristic, we evaluated the disease control rate (DCR), which was regarded as the percentage of patients with partial response or stable disease. All DCRs were summarized in a forest plot. The F-score test was used to evaluate the significant association between a categorical variable and the DCR. A result was considered significant when the *p*-value was less than 0.10, which we consider acceptable given the small sample size.

In order to predict initial chemotherapy response, we used a well-known machine learning method, i.e., random forest (RF). This is an ensemble machine learning classifier that generally provides good performance with low overfitting [26]. RF provides an embedded method for feature selection: it takes advantage of its own feature selection process and performs classification at the same time. In our work, we used the decrease in Gini impurity when a feature is chosen to split a node of a tree and a standard configuration of RF with 100 trees and 20 features, randomly selected at each split, as described by Breiman [27]. Moreover, to control the overfitting risk, we fixed a small number of observations per tree leaf, such as three. More complicated architectures did not give any significant classification improvement.

Missing clinical attribute values were treated according to the predictive value imputation method by replacing missing values with the average of the attribute observed in the training set [28].

Other state-of-the-art classifiers were evaluated, but these did not lead to a significant improvement in performance. These results are not shown to avoid complicating the discussion herein.

The sequential foreword selection algorithm identified a subset of features that best predicted the expected results by sequentially adding features from the initial candidate set until there was no improvement of performance prediction evaluated in terms of area under the curve (AUC) of the receiver operating characteristic (ROC) curve.

The classification performances were evaluated in 100 tenfold cross-validation rounds in terms of AUC, and accuracy, sensitivity, and specificity calculated by identifying the optimal threshold using Youden’s index on the ROC curves [29].

The feature selection was nested in the cross-validation, so at each iteration, the suitable selected subset could change due to the training set. Therefore, at the end of the 100 tenfold cross-validation rounds, we identified the occurrence frequency of the selected features in the first 7 iterations using the sequential foreword procedure in 100 tenfold cross-validation rounds.

Feature importance techniques and classification models were performed using the MATLAB R2021b (Mathworks, Inc., Natick, MA, USA) software.

## 4. Results

Table 1 summarizes the characteristics of the analyzed samples. A total of 46 patients affected by unresectable disease (with a median age of 72 years old, and average first and fourth quartiles of 48 and 86 years, respectively) were evaluated. The histotype was epithelioid in 37 patients, biphasic in 7 patients, and sarcomatoid in 2 patients. TNM stage (8th ed.) was II in 11 patients, III in 23 patients, and IV in 12 patients. A total of 30 patients received standard platinum plus pemetrexed chemotherapy, while 16 patients were treated with innovative treatments in clinical trials (five patients with standard chemotherapy plus bevacizumab, five patients with immunotherapy anti-PDL1 plus anti-CTLA4, four patients with standard chemotherapy plus bevacizumab and anti-PDL1 immunotherapy, and two patients with standard chemotherapy plus anti-PDL1 immunotherapy).

**Figure 1:**
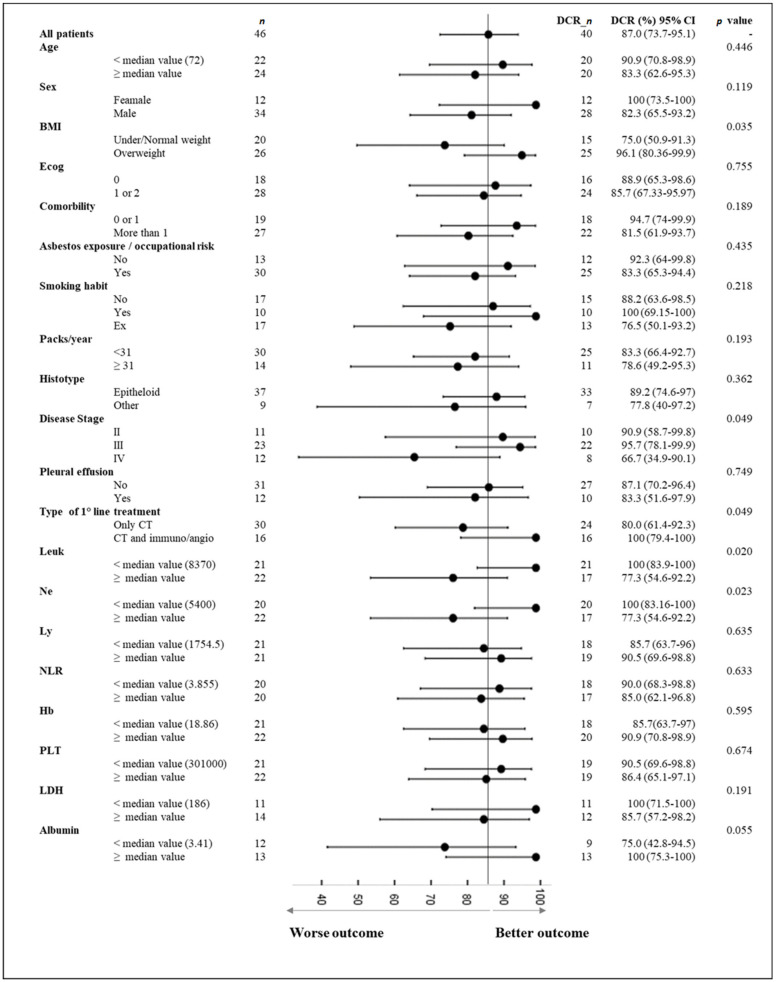
Forest plot of initial disease control rate (DCR) for several characteristics. CI: confidence interval. Abbr.: Leuk: leukocytes; Ne: neutrophils; Ly: lymphocytes; NLR: neutrophil lymphocyte ratio; Hb: hemoglobin; PLT: platelets; BMI: body mass index.

A total of 40 patients showed a therapeutic initial partial response or stable disease upon reassessment. Among the clinical characteristics, our preliminary evaluation showed that, according to the treatment type, the group of patients treated with regimens including antiangiogenic and/or immunotherapy obtained a significantly better initial response (*p*-value 0.049) (Figure 1).

Furthermore, the therapeutic response was significantly correlated with the disease stage (*p*-value 0.049). Specifically, the patients with a tumor stage of III showed a DCR of 95.7% (95% IC, 78.1–99.9%), whereas for disease stage IV, the DCR decreased to 66.7% (95% IC, 34.9–9.1%). Moreover, the therapeutic response was significantly correlated with blood values of leucocytes, neutrophils, and albumin serum levels (*p* values < 0.10). In particular, the initial response to chemotherapy was more favorable for patient with lower levels of leukocytes and neutrophils, and higher albumin serum levels; these patients reached a DCR of 100% (95% IC, 83.90–100%), 100% (95% IC, 83.16–100%), and 100% (95% IC, 75.30–100%), respectively. A better initial response was also observed in overweight patients (*p*-value 0.035), who achieved a DCR of 96.10% (95% IC, 80.36–99.9%).

The proposed method was trained on a subset of significant features identified by means of a sequential forward feature selection algorithm. Specifically, the binary RF classifiers were trained to recognize responders (stable or partial response) from non-responders (disease progression) on a significant feature subset identified by the feature selection process.

**Figure 2:**
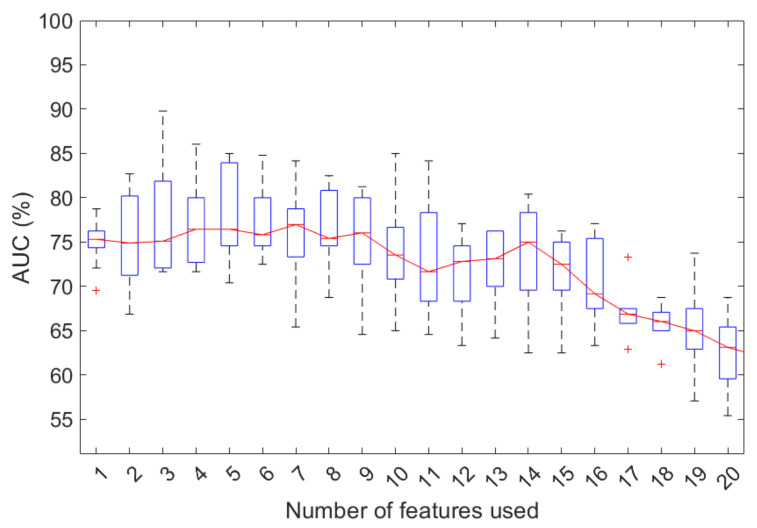
Classification performances to predict the initial response to therapy for an increasing number of features selected by the stepwise forward selection procedure in 100 tenfold cross-validation rounds. +: outliers of the distribution.

The classification performances for an increasing number of features identified by the sequential feature selection algorithm are shown in Figure 2. The model performances seemed particularly variable, perhaps due to the small sample size and the strong imbalance of the training classes. However, the best performances were observed when considering seven features, with a median AUC value of 77.0% (interquartile range, IR of 73.3–78.8%); beyond this value, a reduction in classification performance was observed. Specifically, with seven features, the classification model showed a median accuracy, sensitivity, and specificity of 75% (IR 63.04–84.8%), 74.8% (IR 57.5–87.5%), and 83.3% (IR 667.7–100%), respectively.

Table 2 shows the frequency of occurrence of each feature in the first seven iterations of the forward sequential algorithm in 100 tenfold cross-validation rounds.

The features most frequently selected were gender, histotype, BMI, smoking habits, packs/year, and disease stage.

## 5. Discussion

Taking into account the large number of studies in progress concerning pleural mesothelioma, pending further data about the immune microenvironment [30,31,32], especially with regard to possible biomarkers that are both prognostic and predictive of response to treatment, our intent was to evaluate certain clinical parameters that are useful for guiding therapeutic choices.

This could become particularly useful when innovative treatments are available in clinical practice and more careful selection of patients eligible for new therapeutic options is required. This will also be relevant due to the expected higher cost of new therapies as compared to the current standard options.

Indeed, a better definition of prognosis and predictors of response could influence the therapeutic algorithm for these patients in the near future [31,32,33,34].

To the best of our knowledge, despite the important need for new methods to identify predictive factors of the therapeutic outcome of mesothelioma, the literature is limited in this regard.

Recently, various studies evaluated the possibility of predicting the overall survival of mesothelioma patients on the basis of clinical or radiomic features [35,36,37,38,39].

The aim of our study was to carry out a preliminary exploratory analysis of certain clinical characteristics as predictive of therapeutic initial response; our findings showed that, according to the treatment type, the group of patients treated with chemotherapy combined with antiangiogenics and/or immunotherapy obtained significantly better results.

The therapeutic outcome was also significantly correlated with the disease stage (*p*-value 0.049), the basal BMI, and the serum albumin levels; in particular, patients with stage II–III disease, higher BMI, and higher albumin levels exhibited better responses. According to the obesity paradox in lung cancer, a high body mass index (BMI) is associated with a better outcome [40,41,42].

Furthermore, with the purpose of evaluating the explanatory contribution of the set of collected features in solving the problem of predicting the initial response to therapy, we trained a state-of-the-art machine learning model combined with a sequential feature selection algorithm. With only seven features, the classification model showed a median AUC, accuracy, sensitivity, and specificity of 77.0%, 75.0%, 74.8%, and 83.3%, respectively. The features with greater informational power were gender, histotype, BMI, smoking habits with packs/year, and disease stage.

To the best of our knowledge, the few methods developed for the prediction of treatment outcomes for advanced mesothelioma concern overall survival [35,36]; our work instead aimed to predict the initial response to therapy, attributing a role to long-term outcome, while considering it a useful tool to better select patients eligible for different treatment options. For this reason, we believe that a comparison of performance with survival models is inappropriate.

Although the model can be optimized by evaluating other feature selection techniques or classification algorithms using a more extensive database, in our opinion, clinical characteristics analyzed alone are not sufficient to develop a support tool suitable for current clinical practice.

What emerges, and what is important to emphasize to an audience of biomedical data scientists and clinicians, is that the informative power contained in the characteristics considered in this study for the prediction of the initial response to treatment is not negligible. Hence, we initiated a study concerning the role of radiomic features extracted from pre-treatment CT images encouraged by recent studies with important results regarding the joint use of histopathological and radiomic features, despite them being developed in different research tasks [43,44,45,46,47].

The possible future directions of our findings are manifold. The relatively small size of the dataset represents one of the limitations of this study, but we emphasize that mesothelioma is a rare disease. Moreover, our study is a feasibility study and the advantage of the small sample that we analyzed is that it is homogeneous by stage and systemic treatment; therefore, we believe that, similar to other published studies [48], the group of patients analyzed is congruous with respect to the pathology for a mono-center study.

This allowed us to carry out preliminary assessments without being vitiated by the known biases of multicenter studies. In addition, machine learning algorithms do not necessarily need large numbers for training. However, in order to obtain a measure of the variability of the performance for the test set, we carried out more rounds of cross-validation. We are aware of the validation bias in cross-validation, but various studies show that, if feature selection is engaged in cross-validation, the classification bias is negligible [49,50,51,52]. The generalizability and flexibility of the proposed method can be improved through method validation on larger datasets.

## 6. Conclusions and Future Directions

The clinical response, assessed after the first two courses of therapy, could represent a useful parameter with which to identify patients with a better outcome and to better select patients who could potentially benefit from innovative therapeutic options.

Therefore, we evaluated a novel machine learning approach to predict the initial response to therapy based on clinical data, achieving a moderately accurate model.

These preliminary data in a small number of patients do not allow concrete conclusions to be drawn; nevertheless, this work is the basis of an ongoing study involving an integrated model to predict the initial therapeutic response based on clinical and radiomic features extracted from pre-treatment CT images that, through artificial intelligence, will evaluate the radiological and biological parameters in these patients by integrating them with clinical and histological data.

Using a radiomics-based approach, an important goal is the selection of patients with a better therapeutic outcome, especially with the foreseeable advent of innovative therapies in this field. Finally, as a result of the hypothesized advantage for patients treated with innovative strategies, we believe it is essential to seek the most suitable methods to predict treatment response, in order to better update the prognosis of a rare and insidious neoplasm such as pleural mesothelioma.

## Figures and Tables

**Table 1:** Characteristics of the 46 patients analyzed in the study.

Patient Characteristics		Absolute Value	%
Age	Median (1st-quartile; 3rd-quartile)	72 (48; 86)	
Gender			
	Female	12	26.09
	Male	34	73.91
BMI			
	Under/Normal weight	20	43.48
	Overweight	26	56.52
ECOG			
	0	18	39.13
	1 or 2	28	60.87
Comorbidities			
	0 or 1	19	41.30
	More than 1	27	58.70
Asbestos exposure/occupational risk			
	No	13	28.26
	Yes	30	65.22
	NaN	3	6.52
Smoking habit			
	No/Ex	34	73.91
	Yes	10	21.74
	NaN	2	4.35
Packs/year			
	<31	30	68.18
	≥31	14	31.82
	NaN	2	
Histotype			
	Epithelioid	37	80.43
	Non-epithelioid	9	19.57
Disease stage			
	II	11	23.91
	III	23	50.00
	IV	12	26.09
Pleural effusion			
	No	31	67.39
	Yes	12	26.09
	NaN	3	6.52
Type of 1st line treatment			
	Only CT	30	65.22
	CT plus immuno/angio	16	34.78
Leuk	Median (1st-quartile; 3rd-quartile)	8370 (7030; 11250)	
	Valid value/NaN	43/3	93.47/6.52
Ne	Median (1st-quartile; 3rd-quartile)	5400 (4210; 8002.5)	
	Valid value/NaN	42/4	91.30/8.70
Ly	Median (1st-quartile; 3rd-quartile)	1754.5 (1315; 2172.5)	
	Valid value/NaN	42/4	91.30/8.70
NLR	Median (1st-quartile; 3rd-quartile)	3.855 (1.875; 5.600)	
	Valid value/NaN	40/6	86.96/13.04
Hb	Median (1st-quartile; 3rd-quartile)	12.86 (12.065; 14.00)	
	Valid value/NaN	43/3	93.47/6.52
PLT	Median (1st-quartile; 3rd-quartile)	301000 (234500; 383500)	
	Valid value/NaN	43/3	93.47/6.52
LDH	Median (1st-quartile; 3rd-quartile)	186 (170; 207)	
	Valid value/NaN	24/21	52.17/47.83
Albumin	Median (1st-quartile; 3rd-quartile)	3.41 (2.83; 3.70)	
	Valid value/NaN	24/21	52.17/47.83

Abbr.: Leuk: leukocytes; Ne: neutrophils; Ly: lymphocytes; NLR: neutrophil lymphocyte ratio; Hb: hemoglobin; PLT: platelets; BMI: body mass index.

**Table 2:** Occurrence frequency of the selected features in the first seven iterations using the sequential forward procedure in 100 tenfold cross-validation rounds.

Features	Occurrence Frequency (%)
Gender	54%
Histology	52%
BMI	50%
Smoking habits	44%
Packs/year	41%
Disease stage	40%
Comorbidity	36%
Type of 1st line treatment	32%
Ne	32%
NLR	30%
Asbestos exposure/occupational risk	23%
LDH	20%
Age	15%
Pleural effusion	14%
Leuk	12%
PLT	11%
Albumin	6%
ECOG	4%
Ly	4%
Hb	2%

Abbr.: Leuk: leukocytes; Ne: neutrophils; Ly: lymphocytes; NLR: neutrophil lymphocyte ratio; Hb: hemoglobin; PLT: platelets; BMI: body mass index.

## Data Availability

The data presented in this study are available on request from the corresponding author. The data are not publicly available because are propriety of I.R.C.C.S.—Istituto Tumori ‘Giovanni Paolo II’—Bari, Italy.
